# Improving Outcomes in Alcohol Withdrawal; an Alcohol and Drug Liaison Outreach Approach

**DOI:** 10.1192/bjo.2024.422

**Published:** 2024-08-01

**Authors:** Megan Robertson, Thomas Walker, Lorraine Hope

**Affiliations:** NHS Ayrshire & Arran, Kilmarnock, United Kingdom

## Abstract

**Aims:**

This project's purpose was to improve the identification and management of patients at risk of or suffering from alcohol withdrawal at the point of admission. Ultimately aiming to prevent avoidable harm to patients and reduce the burden on local services within NHS Ayrshire & Arran.

**Methods:**

The project began in August 2023 with Alcohol & Drug Liaison Nurses (ADLN) carrying out twice daily walkthroughs of the Emergency Department and Combined Assessment Unit. ADLNs were instructed to engage with these clinical teams, helping to identify those at risk, provide management advice and accept relevant referrals. A retrospective audit was completed covering patients referred to the alcohol and drug liaison team (ADLT) in July 2023 and a prospective audit covering October 2023. Quantitative data gathered included prescription of benzodiazepines & Pabrinex, time from admission to prescription and administration of treatments, any changes to treatment advised, and whether treatments administered correctly. Additional qualitative data was gathered through a short staff survey carried out in November 2023 asking if the project had been helpful in identifying patients, improving management, and making staff feel supported.

**Results:**

There was a 33% increase in referrals from July (n = 15) to October (n = 20), with a slightly greater proportion coming from ED (80% vs 85%). The average time from admission to benzodiazepine prescription fell by 2hrs and to administration by 8hrs. However, changes were advised to benzodiazepines prescriptions more frequently (12% increase).

Pabrinex prescriptions were being completed overall for patients both before (92%) and after (100%) the project. Average time from admission to pabrinex prescription fell by 2hrs but to administration increased by 0.5hrs. Additionally, cases of incorrect pabrinex administration increased from 31% to 47% between the two periods.

Staff feedback was very positive; project was very (45%) or somewhat (35%) helpful in identifying patients at risk, very (30%) or somewhat (50%) helpful in managing alcohol withdrawal, and very (55%) or somewhat (20%) helpful in making staff feel more supported with this patient group.

**Conclusion:**

This project demonstrated that additional support can improve identification of patients, speed of initial management decisions, and staff confidence. However, it also showed a significant knowledge/skills gap across both departments leading to continued problems with patients receiving timely and appropriate treatment. ADLN ward input cannot be continuous, as such practical changes are required to help maximize Liaison input. Following this project's recommendation, work has begun to develop an Alcohol Withdrawal Bundle and associated staff training.

